# Implementing a Labor and Delivery Cell Salvage Protocol in Patients at Increased Risk of Hemorrhage: A Pilot Study

**DOI:** 10.7759/cureus.73334

**Published:** 2024-11-09

**Authors:** Camila Cabrera, Nicola F Tavella, Cody P Goldberger, Chioma Iwelumo, Eric Mitchell, David Lubell, Angela T Bianco, Daniel Katz, Rebecca H Jessel

**Affiliations:** 1 Maternal-Fetal Medicine, Winnie Palmer Hospital for Women and Babies, Odessa, USA; 2 Obstetrics and Gynecology, Icahn School of Medicine at Mount Sinai, New York City, USA; 3 Obstetrics and Gynecology, New York University Grossman School of Medicine, New York City, USA; 4 Obstetrics and Gynecology, Mount Sinai West Medical Center, New York City, USA; 5 Obstetrics and Gynecology, Mount Sinai Hospital, New York City, USA; 6 Anesthesiology, Mount Sinai Hospital, New York City, USA; 7 Obstetrics and Gynecology, New York University Langone Hospital - Brooklyn, Brooklyn, USA

**Keywords:** alternative transfusion strategies, autologous blood transfusions, blood salvage, obstetric anesthesia, postpartum hemorrhage

## Abstract

Background: Postpartum hemorrhage (PPH) contributes significantly to maternal morbidity and mortality. The use of cell salvage has been implemented in operating rooms across the world, but only a limited number of institutions have protocols for use of cell salvage during vaginal hemorrhage at the time of vaginal delivery. Observations suggest that blood salvaged from vaginal delivery is comparable to blood salvaged during cesarean delivery. Using pre-validated protocols of cell salvage, we sought to assess the feasibility and potential benefit of implementing cell salvage in our Labor and Delivery unit in all patients at high risk of hemorrhage.

Methods: This was a prospective pilot study conducted from April 2022 to December 2022 on the Labor and Delivery floor at Mount Sinai Hospital in New York City. A total of 50 participants were identified for cell salvage after vaginal delivery during the study period. The mean age of participants was 34.4 years (SD 5.5). We utilized a cell salvage technique at the time of vaginal delivery in patients at high risk of PPH. We employed simple descriptive statistics and examined sums and percentages (and means and standard deviations, where appropriate). A simple equation was used to determine the average cell salvaged volume in each delivery and describe potential values. The HEMAsavR™ device (Ecomed Solutions, Mundelein, IL, USA) was used as a standby system to be used at the time of the vaginal delivery.

Results: Fifty participants were identified for the cell salvage protocol as described. Despite a diversity of clinical risk factors, the sample consisted of predominately non-Hispanic White patients. The mean quantitative blood loss of cell salvaged samples was 157.2 mL (SD 153.0). We identified that, on average, >33% of vaginally shed blood could be used for cell salvage and improve patient blood management.

Conclusion: The implementation of cell salvage in our Labor and Delivery unit was feasible and easy to perform. We identified that a significant volume of blood would be available for cell salvage. Further studies should be done to evaluate the benefit of cell salvage to improve postpartum recovery.

## Introduction

Postpartum hemorrhage (PPH) is the leading cause of maternal mortality worldwide, accounting for 27.1% of maternal deaths [1]. PPH accounts for a significant proportion of maternal deaths in the United States (US). It is surpassed only by cardiovascular conditions and sepsis and is the leading cause of death on the day of delivery [2]. The management of PPH has evolved as the medical community attempts to avoid such preventable deaths [3]. Massive transfusion protocols, new mechanical devices, and cell salvage have all been implemented in hemorrhage protocols in the past decade [4]. Mechanisms like cell salvage represent a growing scientific consensus towards patient blood management (PBM) that endeavors to safeguard an individual's own blood supply [5]. Such consensus has produced clinical practice guidelines to improve integrating PBM into clinical care across disciplines [6]. Although cell salvage transfusion at the time of vaginal hemorrhage has been deemed safe, it remains sparsely implemented globally. A recent national study of cell salvage among cases of PPH in Germany across 10 years found a <0.1% rate of cell salvage [7].

Concerns about amniotic fluid embolism with cell salvage in obstetrics have hindered its widespread use [8]. Enhancing the washing process and using leucocyte depletion filters may yield safer implementation and a better benefit-to-risk ratio for use in obstetrics [9]. To date, although limited, studies have suggested that blood salvaged from vaginal and cesarean deliveries contains similarly low quantities of bacteria after it has been washed [10]. In addition, adverse events during the use of cell salvage (i.e., sepsis, wound infection, or thromboembolism) are anecdotally uncommon [11]. However, there remains a paucity of data on cell salvage after vaginal delivery, despite such deliveries representing the majority of US births. Here, we explore whether the application of cell salvage during vaginal deliveries at high risk of hemorrhage was feasible at a single urban tertiary care center.

## Materials and methods

This pilot study was approved by the Institutional Review Board of Icahn School of Medicine at Mount Sinai, and this included a waiver of written consent. The trial was conducted from April 2022 to December 2022 on the Labor and Delivery floor at Mount Sinai Hospital in New York City, USA; approximately 7,000 deliveries occurred at our institution in 2022. This was a prospective study that employed a convenience sample of available high-risk patients. All potential participants were first approached by their primary obstetric provider either at their routine prenatal visits or upon admission to the labor floor. Participants were eligible for the study if they had one or more risk factors for postpartum hemorrhage including a history of PPH, four or more prior deliveries, multiple gestation, large for gestational age fetus, fibroid uterus, polyhydramnios, and prolonged labor course with ≥18 hours of oxytocin administration. Eligible patients who agreed to participation were approached by the study team for verbal consent.

**Figure 1:**
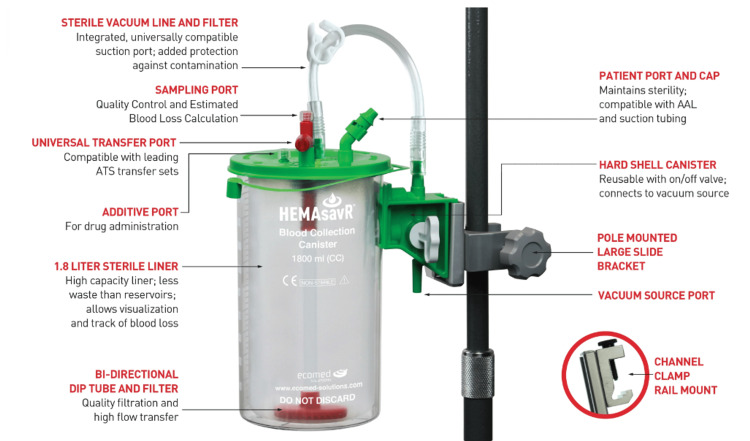
HEMAsavR™ canister for cell salvage during labor and delivery ATS, autotransfusion system; AAL, autologous anticoagulated line Image is courtesy of Ecomed Solutions (Mundelein, IL, USA).

Following the technique described by Phillips et al., we set up the HEMAsavR™ canister (Ecomed Solutions, Mundelein, IL) as a standby system to be used at the time of the vaginal delivery [9,11]. This system consists of a reusable hard-shell canister with a sterile, self-contained, and disposable inside liner with attachments that facilitate transfer of the salvaged blood into the cell saver reservoirs (Figure 1).

**Figure 2:**
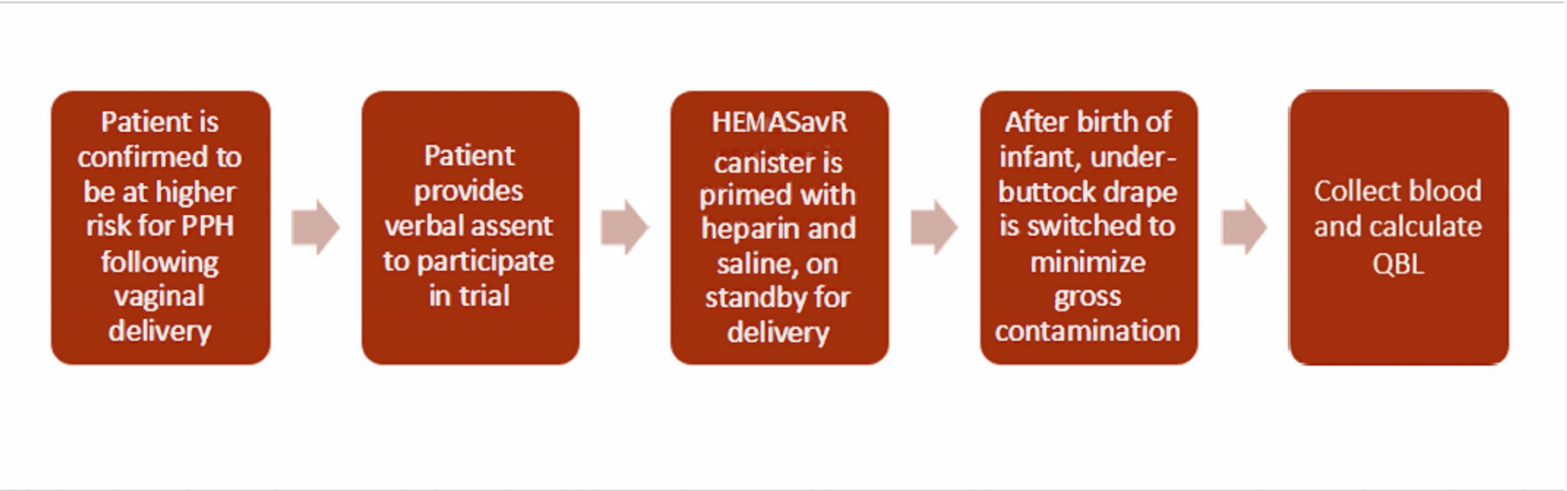
Flow diagram of enrolling subjects into the study and conducting cell salvage during labor and delivery PPH, postpartum hemorrhage; QBL, quantitative blood loss Image is an original product of the authorship team.

Study team members were trained on the set-up and use of the device. Prior to the study, labor and delivery nurses received in-service education about the HEMAsavR device, the blood collection protocol, and autotransfusion methodology. Canisters were primed with 10,000 units of heparin and 100 mL of saline in preparation for the collection of blood. We found that preparing the canisters with tubing, heparin and saline as well as preparing the delivery table with two under-buttock drapes and a Yankauer tip made implementation more efficient at delivery. We were cautious to set the suction no higher than 200 mmHg to avoid lysis of the red blood cells. Once the umbilical cord was clamped and cut, a clean under-buttock drape was placed under the patient with the goal to minimize gross contamination from the suction field, and blood collection was started (Figure 2). A total of 50 salvage devices were primed and used, with zero re-infusions.

We employed simple descriptive statistics to describe study results. We examined sums and percentages (and means and standard deviations, where appropriate) and used a simple equation (mentioned below) to determine the average cell salvaged volume in each delivery and describe potential values found:

$$\frac{\text{Average hematocrit level of the cell salvage sample}}{\text{Average quantity of blood salvaged}} = \frac{\text{0.55}}{250 mL}.$$

We used the known hematocrit level of 55% for a 250 mL unit of cell salvaged blood to calculate the average quantity (mL) salvaged in our trial using hematocrit values from samples collected [12]. We used a GEM 5000 blood gas analyzer (Werfen, Bedford, MA) to obtain the hematocrit values of the collected blood samples. This yielded an estimated quantity of blood salvaged at each delivery through this protocol.

## Results

Fifty participants were identified for cell salvage after vaginal delivery during the study period. Table 1 displays the demographics, eligibility criteria, and baseline laboratory values of the sample.

**Table 1:** Demographics of the study sample PPH, postpartum hemorrhage ^a^Total N = 50

Characteristic	N (%)/mean (SD)^a^
Age at delivery (mean)	34.4 (5.5)
Gestational age at delivery (mean)	38.8 (1.5)
Gravidity (mean)	7.7 (3.3)
Parity (mean)	5.5 (2.5)
Race and ethnicity	
Non-Hispanic White	49 (98)
Non-Hispanic Black	1 (2)
Insurance coverage	
Private	18 (36)
Public	32 (64)
Comorbidities	
Chronic hypertension	2 (4)
Pregestational diabetes	1 (2)
Chronic anemia	5 (10)
Cardiovascular disease	3 (6)
History of PPH	7 (14)
None	37 (74)
Obstetric complications	
Gestational diabetes	3 (6)
Severe preeclampsia	2 (4)
None	45 (90)

The mean age of participants was 34.4 years (SD 5.5). The sample included one non-Hispanic Black participant and 49 non-Hispanic White participants. Thirty-two (65%) participants had public insurance. Thirty-seven (74%) participants had no comorbidities at delivery, 7 (14%) had a prior history of PPH, 5 (10%) had diagnosed anemia at the time of delivery, 3 (6%) had cardiovascular disease, 2 (4%) had chronic hypertension, and 1 (2%) had pregestational diabetes. While 45 (90%) participants had no obstetric complications, 3 (6%) participants developed gestational diabetes and 2(4%) developed severe preeclampsia. The average gestational age at the time of delivery was 38.8 weeks (SD 1.5). The average gravidity was 7.7 (SD 3.3), and the average parity was 5.5 (SD 2.5).

The amount of saline used to prime the canister evolved over time in this pilot study of using the HEMAsavR collection system after vaginal delivery. A volume of 100 mL of saline was consistently used one month after pilot study initiation as a volume recognized to facilitate set-up, calculation of quantitative blood loss (QBL), and measure hematocrit. The mean volume of saline suctioned over the study period was 107.8 mL (SD 94.3). The calculation of QBL was possible in 44 of 50 cases. For six cases in which QBL could not be calculated, reasons included suction malfunction (n=2), precipitous delivery missed (n=2), arrest of dilation (n=1), and QBL too low (n=1). The mean QBL of cell salvaged samples was 157.2 mL (SD 153.0). The institutional protocol includes the administration of oxytocin in all patients after delivery. The administration of additional uterotonics at the preference of the obstetrical provider occurred for 40 (80%) participants: 31 (62%) participants received Methergine, 3 (6%) participants received Hemabate (carboprost), and 6 (12%) participants received misoprostol. In our study population, there were seven cases of PPH (QBL >500 mL for a vaginal delivery) with a maximum QBL of 950 mL. Hematocrit averaged 11.76% (SD = 7.7) in 31 cases. Using this average hematocrit value and assuming that a typical unit of cell salvaged blood has a hematocrit level of 55% and a volume of 250 mL, we calculated the mean volume of blood salvaged to be 53.5 mL.

## Discussion

We identified that, on average, 21.4% of a unit of blood could be salvaged in deliveries where vaginally shed blood could be collected even in cases without PPH. Despite most deliveries being vaginal, there remains little generalizable data on the use of cell salvage in vaginal deliveries. The HEMAsavR™ technology is likely most beneficial in cases where the provider encounters an ongoing hemorrhage, those with anticoagulation use or a known coagulopathy, or patient objection to an allogenic blood transfusion. We hypothesize that HEMAsavR™ and subsequent cell salvage transfusion would be helpful as well in cases of maternal antepartum anemia. Anemia complicates approximately 40% of pregnancies worldwide, with the prevalence of anemia among pregnant women in the United States steadily increasing [13]. Postpartum anemia affects 50%-80% of patients, and has been linked to symptoms like fatigue, breathlessness, palpitations, and urinary tract infections [14]. Furthermore, postpartum anemia is associated with an increased risk of postpartum depression and impaired cognition and has been shown to negatively impact maternal-infant bonding [15,16]. The importance of combating antepartum anemia and engaging PBM protocols cannot be overstated, as this provides the first line of clinical support ahead of potential PPH [5,6]. The assessment of full postpartum iron status should inform further efforts; we recommend blood transfusion as most efficacious, but intravenous iron can also replenish iron stores adequately relative to oral iron supplementation.

Although the study team found the implementation process of the HEMAsavR™ device to be feasible, an additional team member was required at the time of delivery to ensure that the delivery drape was removed promptly and then suction blood collected into the second v-drape while the primary provider actively managed the third stage of labor and laceration repair. In the setting of hemorrhage, additional personnel enter the room to assist and one of these assistants can be assigned to cell salvage, but in the setting of routine vaginal delivery, there may not be a dedicated person to perform suctioning. A major limitation of the study protocol and the broader use of cell saver is the cost and effort of additional personnel. Additionally, the use of cell salvage remains understudied in the literature; hence, a clear safety profile has not yet been established to encourage widespread adoption. Finally, the present study is limited by its nature as a pilot investigation: (1) a small sample size reduces the statistical power to detect significant associations, (2) the small single-site environment and a short trial period limit the generalizability of findings to long-term trends in broader populations, and (3) the potential for bias, especially selection bias, is high, questioning the representativeness of the study population.

However, several conclusions can be drawn from our pilot study assessing the feasibility and potential benefit of implementing cell salvage technology in patients at high risk of hemorrhage after vaginal delivery. We found the implementation of the blood collection system to be feasible and easy to perform in our Labor and Delivery unit. However, we conclude that the use of the blood collection system in every high-risk vaginal delivery may not be time efficient or cost-effective. Although vaginal delivery is the predominant mode of delivery accounting for 70% of all births in the United States, PPH remains rare. Previous studies have examined the cost of allogenic blood transfusions and found an estimated cost of US$1,178 for two units of transfused red blood cells [17]. When considering this cost relative to the prospect of a catastrophic health expenditure, which is more likely during the labor and delivery process, preparation of cell salvage is certainly cost-effective [18]. However, given that none of the 50 participating patients in this pilot study had excess bleeding at the time of delivery despite their various risk factors for hemorrhage, we can surmise that it is not cost-effective to open a disposable HEMAsavR™ sleeve for each high-risk patient. The presence of the HEMAsavR™ device on standby in these delivery rooms allows for its use only in settings where sufficient blood collection can be guaranteed to successfully fill a cell saver reservoir, while minimizing the delay in sterile and anticoagulated blood collection when needed, as well as the waste of resources. Therefore, it may be cost-effective to have the canister and necessary accessories in each room as standby units if a hemorrhage is identified during delivery.

## Conclusions

In this study, we examined the implementation of cell salvage technology in vaginal deliveries anticipated to be at an increased risk of hemorrhage. Cell salvage in vaginal deliveries is complicated logistically and clinically, yet our study suggests it is a possible method of collection for transfusion. Our pilot study did not yield significant quantities to necessarily impact clinical outcomes; however, we provide a framework onto which clinicians can build improved methods of cell salvage to integrate PBM into institutional delivery practices.

## References

[1] Corbetta-Rastelli CM, Friedman AM, Sobhani NC, Arditi B, Goffman D, Wen T (2023). Postpartum hemorrhage trends and outcomes in the United States, 2000-2019. Obstet Gynecol.

[2] Say L, Chou D, Gemmill A (2014). Global causes of maternal death: a WHO systematic analysis. Lancet Glob Health.

[3] Teare KM, Sullivan IJ, Ralph CJ (2015). Is cell salvaged vaginal blood loss suitable for re-infusion?. Int J Obstet Anesth.

[4] Waters JH, Beck S, Yazer MH (2019). How do I perform cell salvage in obstetrics?. Transfusion.

[5] Spahn DR, Muñoz M, Klein AA, Levy JH, Zacharowski K (2020). Patient blood management: effectiveness and future potential. Anesthesiology.

[6] Mueller MM, Van Remoortel H, Meybohm P (2019). Patient blood management: recommendations from the 2018 Frankfurt Consensus Conference. JAMA.

[7] Neef V, Friedrichson B, Jasny T (2024). Use of cell salvage in obstetrics in Germany: analysis of national database of 305 610 cases with peripartum haemorrhage. Br J Anaesth.

[8] Rogers WK, Wernimont SA, Kumar GC, Bennett E, Chestnut DH (2013). Acute hypotension associated with intraoperative cell salvage using a leukocyte depletion filter during management of obstetric hemorrhage due to amniotic fluid embolism. Anesth Analg.

[9] Phillips JM, Waters J, Larkin J, Tamura T, Sakamoto S (2021). Autotransfusion of vaginally shed blood during obstetric hemorrhage: a matched cohort study. Am J Obstet Gynecol.

[10] Goucher H, Wong CA, Patel SK, Toledo P (2015). Cell salvage in obstetrics. Anesth Analg.

[11] Phillips JM, Tamura T, Waters JH, Larkin J, Sakamoto S (2022). Autotransfusion of vaginally shed blood as a novel therapy in obstetric hemorrhage: a case series. Transfusion.

[12] Campbell-Lee SA, Ness PM (2007). Chapter 18 - Packed red blood cells and related products. Blood Banking and Transfusion Medicine: Basic Principles & Practice (Second Edition).

[13] Kanu FA, Hamner HC, Scanlon KS, Sharma AJ (2022). Anemia among pregnant women participating in the Special Supplemental Nutrition Program for Women, Infants, and Children — United States, 2008-2018. CDC Morb Mortal Wkly Rep.

[14] Milman N (2011). Postpartum anemia I: definition, prevalence, causes, and consequences. Ann Hematol.

[15] Wassef A, Nguyen QD, St-André M (2019). Anaemia and depletion of iron stores as risk factors for postpartum depression: a literature review. J Psychosom Obstet Gynaecol.

[16] Murray-Kolb LE, Beard JL (2009). Iron deficiency and child and maternal health. Am J Clin Nutr.

[17] Glenngård AH, Persson U, Söderman C (2005). Costs associated with blood transfusions in Sweden - the societal cost of autologous, allogeneic and perioperative RBC transfusion. Transfus Med.

[18] Peterson JA, Albright BB, Moss HA, Bianco A (2022). Catastrophic health expenditures with pregnancy and delivery in the United States. Obstet Gynecol.

